# Radioactive cesium accumulation in freshwater fishes after the Fukushima nuclear accident

**DOI:** 10.1186/2193-1801-3-479

**Published:** 2014-08-28

**Authors:** Takaomi Arai

**Affiliations:** Institute of Oceanography and Environment, Universiti Malaysia Terengganu, 21030 Kuala Terengganu, Terengganu Malaysia

**Keywords:** Fukushima nuclear accident, Radioactive contamination, Cesium, Terrestrial environment, Global deposition

## Abstract

The Fukushima Daiichi Nuclear Power Plant (F1NPP) accident released large amounts of radioactive substances into the environment and contaminated the terrestrial and marine ecosystems in East Japan. The unpredicted nuclear accident is of global concern for human health and ecosystems. Investigations of radionuclides in the local environments were performed shortly after the accident began; however the temporal and spatial effects and variations in the released radionuclides on the natural environment remain unclear. In the present study, three-year (May 2011 to March 2014) fluctuations and accumulations of total Cs, ^134^Cs and ^137^Cs in freshwater fishes in Fukushima prefecture after the F1NPP accident were examined. The total Cs, ^134^Cs and ^137^Cs concentrations decreased gradually during the three-year period that followed the F1NPP accident. However higher levels, i.e., exceeding 100 Bq kg^-1^, which is the interim limit of radiocesium level in Japan, were detected in several fish species. Radiocesium accumulation patterns in fishes in Fukushima prefecture varied between regions and corresponded to the environmental radiocesium levels in the Fukushima region. These radionuclides are widely distributed and remain in the natural environment. Moreover, a fresh input of radiocesium substances from the F1NPP site into the terrestrial environment remains.

## Introduction

A catastrophic earthquake and tsunami occurred on March 11, 2011, which caused destruction in northeastern Japan and severely damaged the Fukushima Daiichi Nuclear Power Plant (F1NPP). The loss of power and the subsequent overheating, meltdowns, and hydrogen explosions at the F1NPP site resulted in airborne fallout over the land and the ocean that peaked in mid-March (Chino et al. 2011; Morino et al. 2011; Yasunari et al. 2011). The concentration of radionuclides reached a maximum in mid-April of 2011; however previous work has reported that the artificial release of radionuclides from the F1NPP site has continued (Kanda 2013). The F1NPP accident released a large amount of artificial radioactive fission products such as ^131^I, ^134^Cs, ^137^Cs, ^239^Pu and ^240^Pu from the nuclear reactors into the ambient environment. The uptake of such radionuclides into human bodies is of serious concern (Travnikova et al. 2004; Rainbow 2007; Smith et al. 2009). Therefore, monitoring the long-term behaviour of radionuclides in the environment is an important issue for estimating possible radiological consequences and associated risks (Pröhl et al. 2006).

Among these radioactive fission products, the effects of ^134^Cs and ^137^Cs are the most serious because of their high concentrations and long decay periods (Folsom et al. 1968; Folsom and Grismore 1970; Young et al. 1975) and high bioavailability (Whicker and Schultz 1982). The physical half-lives for ^134^Cs and ^137^Cs are 2.07 years and 30.07 years, respectively. Because cesium and potassium have similar chemical characteristics to alkali elements, radioactive cesium can easily enter into the food chain and become an important contributor to internal radiation dosages in animal bodies, especially in muscle tissues (Peters et al. 1999; Baudin 2000; Leung and Shang 2003; Malek et al. 2004).

Fish are a major source of protein and nutrition; therefore, these species become a potential carrier of radionuclides from the aquatic environment to humans. Because radiocesium (i.e., ^134^Cs, ^137^Cs and the total Cs) accumulates and concentrates in muscle tissues, the consumption of contaminated fish can be an important pathway for human exposure (Forseth et al. 1991; Whicker et al. 1993). Therefore, it is prudent to determine the Cs levels in different fish species to document the prevalence of radiocesium in fish and to account for the transfer of radiocesium to humans through fish. Determining the Cs levels in fish is also important for evaluating the potential use of contaminated areas and the possible effectiveness of remediation activities. In the case of the Chernobyl Accident, the bioaccumulation of radionuclides including radiocesium in fish has been previously studied in Europe (Hakanson et al. 1989; Elliott et al. 1992; Ugedal et al. 1995). Most of the attention was focused on Belarus, the Russian Federation and Ukraine because the water bodies in these areas exhibited higher contamination levels (Mizuno and Kubo 2013). However, in the case of the F1NPP accident, little information is available on radiocesium contamination and accumulation in the local freshwater ecosystems.

Several agencies have conducted investigations to monitor the radioactive characteristics of water, soil, air and biota after the F1NPP accident; considerable data have been reported to understand the present status of radionuclide pollution levels (Buesseler et al. 2011, 2012; Chino et al. 2011; Masson et al. 2011). The Fisheries Agency of the Japanese Government is also frequently publishing the radiocesium data; these data are primarily obtained from fish samples collected since the F1NPP accident (The Fisheries Agency of the Japanese Government 2014a). Although several datasets have been published and are freely accessible, few systematic and comprehensive analyses have been performed to understand the variations and distribution of radiocesium levels in the Fukushima area. It has been three years since the F1NPP disaster; however, the radionuclide pollution issues have not yet converged. In such a situation, systematic and comprehensive investigations of radionuclides in natural environments are required.

In this study, Radiocesium (i.e., ^134^Cs, ^137^Cs and the total Cs) levels in 14 freshwater species from Fukushima prefecture were examined using 1007 samples collected between May 2011 and March 2014. The temporal and spatial variations of each radiocesium isotope gradually decreased during the three-year peiord. However, the present study indicates that the accumulation patterns of radiocesium in freshwater species were different among the studied species.

## Material and methods

**Figure 1:**
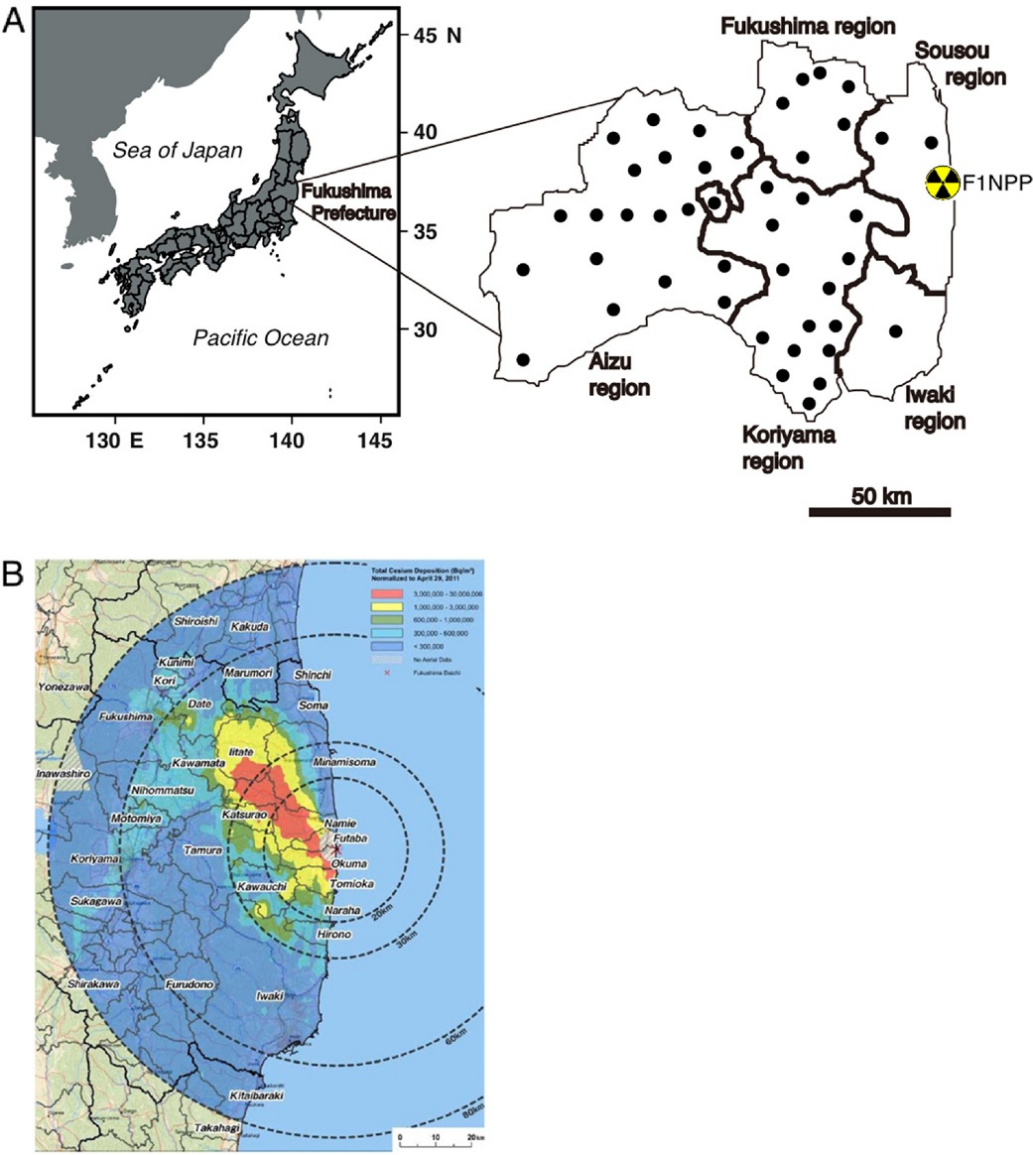
**Study sites and horizontal distribution of radiocesium.** Map showing the location of the study sites in Fukushima prefecture **(A)** and the deposition of radiocesium (i. e. the sum of ^134^Cs and ^137^Cs) for the land area within 80 km of the F1NPP site, as reported by the Japanese authorities (MEXT) **(B)**. Fukushima prefecture has five regions, i.e. the Sousou, Iwaki, Fukushima, Koriyama and Aizu regions, which are divided by thick lines on the map. Closed symbols indicate the location where samples were collected. The map of deposition of radiocesium deposition **(B)** was downloaded from http://www.iaea.org/newscenter/news/2011/fukushimafull.html on 2 June 2014.

In this study, 14 freshwater fish species were examined from 44 sites in five regions of Sousou, Iwaki, Fukushima, Koriyama and Aizu in Fukushima prefecture, Japan, between May 2011 and March 2014 (The Fisheries Agency of the Japanese Government 2014a) (Figure 1 and Table 1). All radiocesium data were from the information published by the Fisheries Agency of the Japanese Government (The Fisheries Agency of the Japanese Government 2014b). With respect to the total number of data sets examined in the total Cs, ^134^Cs and ^137^Cs, 1007, 483 and 696 datasets were examined in this study, respectively. The total Cs concentrations were available between May 2011 and March 2012 in all regions; the radiocesium isotope concentrations were available for all regions during the other periods except for the Sousou region.

**Table 1:** **Radiocesium data information for freshwater fishes examined between May 2011 and March 2014 in the present study**

Species	Number of analysed betweeen May 2011 and March 2012	Number of data above detection limit betweeen May 2011 and March 2012 (Ratio of detectable (%))			Number of analysed betweeen April 2012 and March 2013	Number of data above detection limit betweeen April 2012 and March 2013 (Ratio of detectable (%))			Number of analysed betweeen April 2013 and March 2014	Number of data above detection limit betweeen April 2013 and March 2014 (Ratio of detectable (%))		
		Total Cs*	^134^Cs	^137^Cs		Total Cs*	^134^Cs	^137^Cs		Total Cs*	^134^Cs	^137^Cs
*Plecoglossus altivelis*	74	68	n/a	n/a	59	29	24	28	49	24	15	24
(ayu)		(91.9)				(49.2)	(40.7)	(47.5)		(49.0)	(30.6)	(49.0)
*Salvelinus leucomaenis*	44	41	n/a	n/a	161	113	89	110	177	129	68	127
(white-spotted char)		(93.2)				(70.2)	(55.3)	(68.3)		(72.9)	(38.4)	(71.8)
*Tribolodon hakonensis*	46	43	n/a	n/a	64	55	33	54	75	39	16	39
(Japanese dace)		(93.5)				(85.9)	(51.6)	(84.4)		(52.0)	(21.3)	(52.0)
*Anguilla japonica*	3	2	n/a	n/a	3	3	3	3	2	2	2	2
(Japanese eel)		(66.7)				(100)	(100)	(100)		(100)	(100)	(100)
*Carassius langsdorfii*	18	2	n/a	n/a	13	13	13	13	17	16	14	16
(Japanese silver crucian carp)		(11.1)				(100)	(100)	(100)		(94.1)	(82.4)	(94.1)
*Carassius cuvieri*	3	3	n/a	n/a	1	1	1	1	2	2	2	2
(Japanese white crucian carp)		(100)				(100)	(100)	(100)		(100)	(100)	(100)
*Cyprinus carpio*	13	11	n/a	n/a	23	19	16	19	17	16	10	16
(common carp)		(84.6)				(82.6)	(69.6)	(82.6)		(94.1)	(58.8)	(94.1)
*Micropterus dolomieu*	5	5	n/a	n/a	n/a	n/a	n/a	n/a	n/a	n/a	n/a	n/a
(smallmouth bass)		(100)										
*Misgurnus anguillicaudatu*	4	4	n/a	n/a	1	1	0	1	1	0	0	0
(weather loach)		(100)				(100)	(0)	(0)		(0)	(0)	(0)
*Hemibarbus barbus*	2	2	n/a	n/a	n/a	n/a	n/a	n/a	n/a	n/a	n/a	n/a
(barbel steed)		(100)										
*Oncorhynchus mykiss*	1	0	n/a	n/a	1	0	0	0	n/a	n/a	n/a	n/a
(rainbow trout)		(0)				(0)	(0)	(0)				
*Oncorhynchus nerka*	6	6	n/a	n/a	10	10	10	10	17	17	17	17
(sockeye salmon)		(100)				(100)	(100)	(100)		(100)	(100)	(100)
*Oncorhynchus masou*	69	63	n/a	n/a	120	89	68	89	145	89	51	89
*(masu salmon)*		(91.3)				(74.2)	(56.7)	(74.2)		(61.4)	(35.2)	(61.4)
*Hypomesus nipponensis*	39	39	n/a	n/a	29	26	25	26	13	11	6	11
(pond smelt)		(100)				(89.7)	(86.2)	(89.7)		(84.6)	(46.2)	(84.6)

*Total Cs = ^134^Cs + ^137^Cs.

Total Cs was available between May 2011 and March 2014.

^134^Cs + ^137^Cs were available between April 2012 and March 2014.

n/a: not applicable.

To monitor radiocesium levels in fish samples, the Fisheries Agency in Japan instructed local governments and other organizations to collect samples that are at least 5 kg for each fish species for further radiocesium analyses (The Fisheries Agency of the Japanese Government 2014a). The edible part (muscle) was usually used for measured. The present datasets have also followed this protocol. Radioactive cesium concentration in the fish samples were determined using either a gamma ray spectrometry radionuclide assay method that utilises a germanium semiconductor detector or the NaI scintillation spectrometer method (The Fisheries Agency of the Japanese Government 2014a). The credibility of radiocesium analyses was monitored by the daily measurement of background radiation and the periodic use of a standard radiation source for calibration. All data are depicted below according to the wet weight basis. The detection limit was usually < 10 Bq kg^-1^.

Each radiocesium isotope concentration for each region and in each year was compared using the Kruskal-Wallis test. The significance of the correlation coefficient and the regression slope were determined using a t-test.

## Results

### Temporal variation in radiocesium concentrations

**Figure 2:**
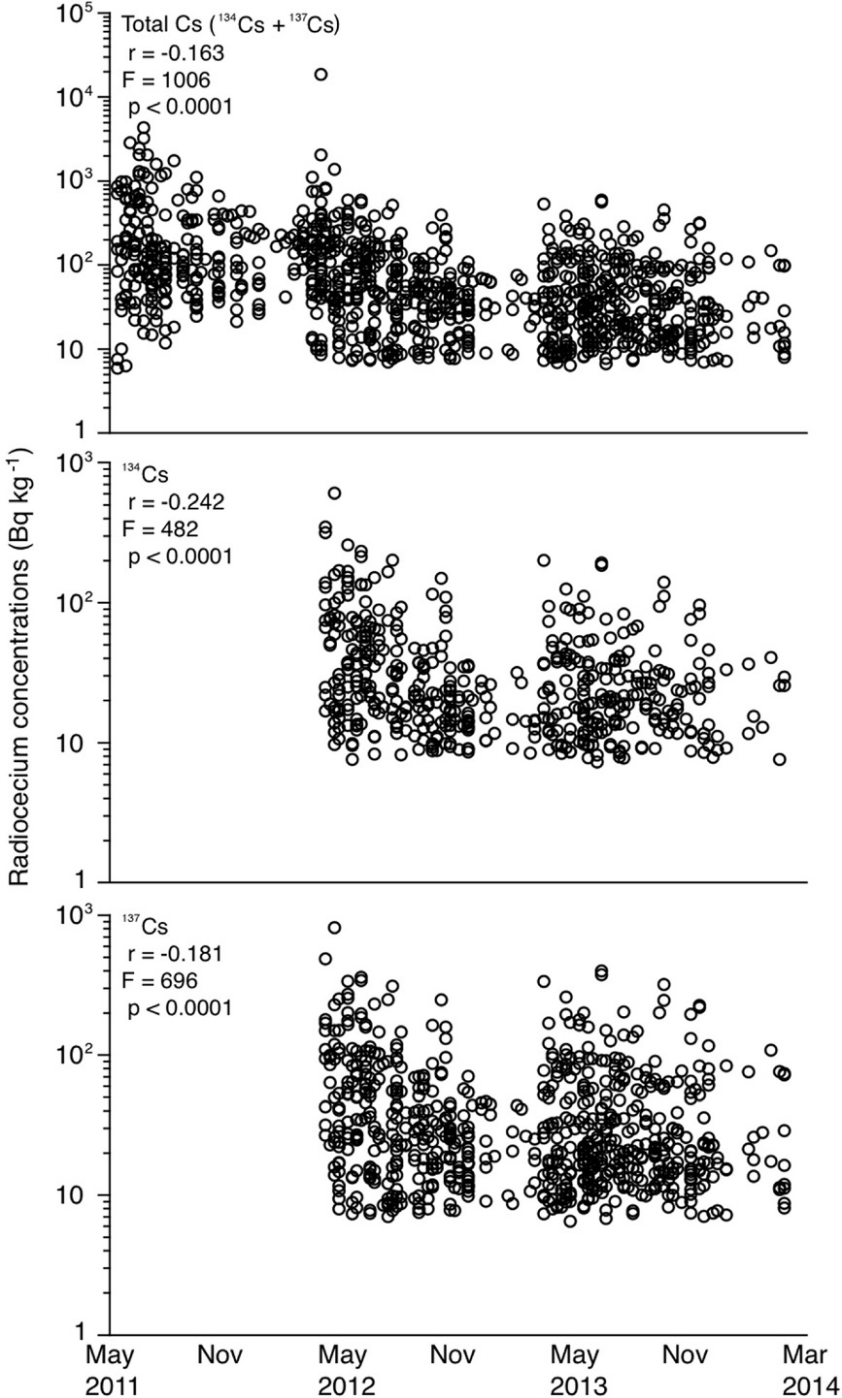
**Variations in the total radiocesium concentrations (top) between May 2011 and March 2014, the** ^**134**^ **Cs (middle) and** ^**137**^ **Cs (bottom) concentrations between April 2012 and February 2014.** The total Cs, ^134^Cs and ^137^Cs concentrations decreased significantly during the three years after the F1NPP accident; however, high concentrations exceeded the 100 Bq kg^-1^ interim limit for radiocesium in Japan were detected. All of the radiocesium data for the salmonids were from the information published by the Fisheries Agency of the Japanese Government (2014a) between May 2011 and March 2014.

**Figure 3:**
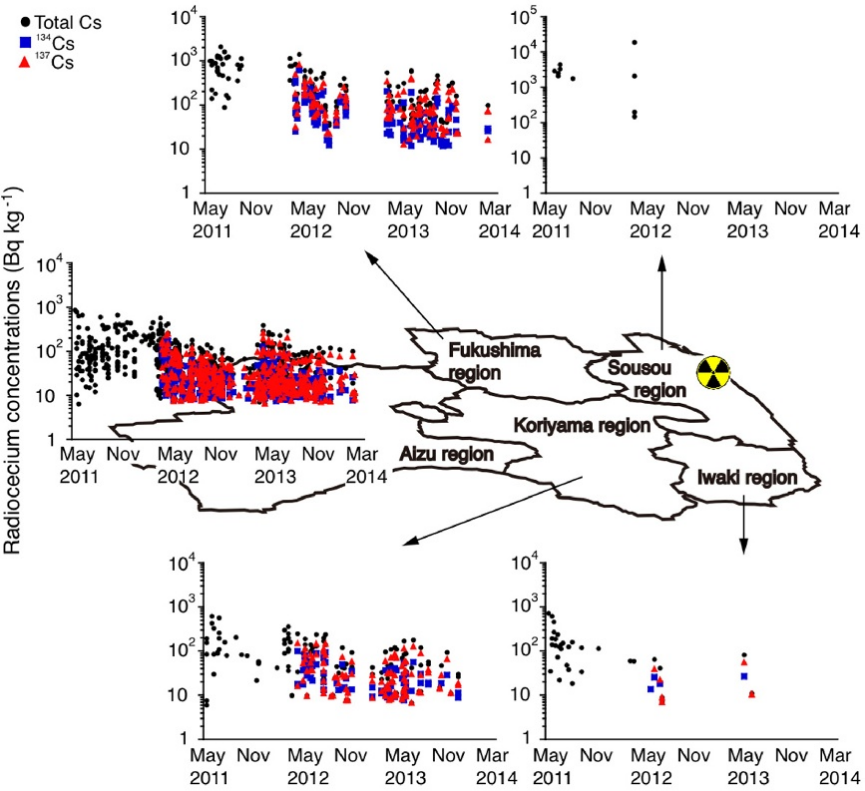
**Temporal and spatial variations in the radiocesium concentrations for three years in five regions in Fukushima prefecture.** Among the five regions in Fukushima Prefecture, almost all sites exhibited a gradual decrease in each radiocesium isotope concentration during the three-year period.

The total Cs, ^134^Cs and ^137^Cs concentrations significantly decreased during the three-year period following the F1NPP accident (F = 482-1006, p < 0.0001) (Figure 2). The highest total Cs concentration (18700 Bq kg^-1^) was found in masu salmon in March 2012 from the Sousou region near the F1NPP site (Table 2 and Figure 3); such a high concentration was not found between April 2012 and March 2014. Although each radiocesium isotope concentration decreased gradually, there were still detectable amounts in freshwater fish in Fukushima prefecture that exceeded 100 Bq kg^-1^ (i.e. the Japanese safety limit) of ^134^Cs and ^137^Cs even three years after the F1NPP accident (Figure 2 and Table 2). The detection ratios of each radiocesium isotope in ayu, white-spotted char, Japanese dace, Japanese silver crucian carp, common carp, masu salmon and pond smelt in which at least 10 samples could be analysed over three-year period also decreased (Table 1). These results suggest that radiocesium accumulated in freshwater fishes in the regions surrounding Fukushima after the F1NPP accident.

**Table 2:** **Radiocecium concentrations (exceeding the detection limit) in freshwater fishes examined between May 2011 and March 2014 in the present study**

Species	Mean ± SD (Bq/kg) between May 2011 and March 2012 (minimum-maximum (Bq/kg))			Mean ± SD (Bq/kg) between April 2012 and March 2013 (minimum-maximum (Bq/kg))			Mean ± SD (Bq/kg) between April 2013 and March 2014 (minimum-maximum (Bq/kg))			
	Total Cs*	^134^Cs	^137^Cs	Total Cs*	^134^Cs	^137^Cs	Total Cs*	^134^Cs	^137^Cs	
*Plecoglossus altivelis*	530 ± 822	n/a	n/a	60.9 ± 61.0	29.1 ± 25.2	37.9 ± 35.5	50.9 ± 42.3	22.8 ± 12.4	36.6 ± 28.4	
(ayu)	7.7-4400			8.5-280	8.4-116	7.58-164	7.9-200	11.8-61.3	7.9-140	
*Salvelinus leucomaenis*	154 ± 145	n/a	n/a	94.2 ± 127	46.3 ± 54.2	59.3 ± 76.1	49.6 ± 75.1	26.2 ± 29.6	36.1 ± 50.4	
(white-spotted char)	6.4-590			7.4-840	7.7-350	7.4-490	6.5-600	7.4-194	6.5-403	
*Tribolodon hakonensis*	207 ± 405	n/a	n/a	75.4 ± 103	45.9 ± 46.5	48.3 ± 61.0	48.1 ± 78.1	32.2 ± 33.3	34.8 ± 51.5	
(Japanese dace)	11-2500			7.8-420	8.8-171	7.8-253	7.1-390	7.9-126	7.1-261	
*Anguilla japonica*	129 ± 21	n/a	n/a	191 ± 179	74.7 ± 69.8	114 ± 106				
(Japanese eel)	114-143			44-390	16.4-152	27.5-82.3	58-110	21.4-34.9	36.2-78.6	
*Carassius langsdorfii*	86 ± 40	n/a	n/a	94.7 ± 91.7	37.0 ± 38.6	57.3 ± 52.2	94.5 ± 91.0	34.8 ± 30.1	64.3 ± 61.7	
(Japanese silver crucian carp)	43-188			24-310	8.6-128	11.2-177	8.9-310	8.9-112	8.9-205	
*Carassius cuvieri*	29 ± 202	n/a	n/a	170	64.1	103				
(Japanese white crucian carp)	88-98						67-120	20.6-38.6	45.9-85.3	
*Cyprinus carpio*	80 ± 46	n/a	n/a	66.9 ± 66.7	31.8 ± 27.8	40.0 ± 39.1	49.3 ± 36.2	22.0 ± 10.3	35.8 ± 24.1	
(common carp)	15-155			13-280	9.4-114	9.9-166	9.6-110	7.8-37.7	9.6-76.6	
*Micropterus dolomieu*	118 ± 130	n/a	n/a	n/a	n/a	n/a	n/a	n/a	n/a	
(smallmouth bass)	10.2-330									
*Misgurnus anguillicaudatu*	49.7 ± 25.6	n/a	n/a	9.7	nd	nd	nd	nd	nd	
(weather loach)	21.6-83									
*Hemibarbus barbus*		n/a	n/a	n/a	n/a	n/a	n/a	n/a	n/a	
(barbel steed)	83-110									
*Oncorhynchus mykiss*	nd	n/a	n/a	nd	nd	nd	n/a	n/a	n/a	
(rainbow trout)										
*Oncorhynchus nerka*	106 ± 42.1	n/a	n/a	136 ± 32.2	51.1 ± 15.7	85.5 ± 18.7	112 ± 27.6	34.1 ± 10.8	77.6 ± 17.7	
(sockeye salmon)	36-158			89-200	34.2-82	55.2-120	81-170	21.8-55.5	54.4-110	
*Oncorhynchus masou*	586 ± 2360	n/a	n/a	116 ± 199	59.2 ± 92.0	71 ± 117	70 ± 101	35.9 ± 36.1	49.4 ± 68.4	
*(masu salmon)*	6-18700			7.1-1430	8.9-610	7.1-820	6.8-570	7.7-187	6.8-378	
*Hypomesus nipponensis*	307 ± 167	n/a	n/a	48.2 ± 18.2	18.7 ± 6.1	30.2 ± 12.2	32.7 ± 19.2	16.7 ± 6.2	23.6 ± 10.0	
(pond smelt)	27-870			9.9-76	8.5-31.8	9.9-46.9	14-76	11.7-28.6	14.4-47.6	

*Total Cs = ^134^Cs + ^137^Cs.

Total Cs was available between May 2011 and March 2014.

^134^Cs + ^137^Cs were available between April 2012 and March 2014.

n/a: not applicable.

nd: All samples were below detection limit.

### Spatial variations in radiocesium concentrations

Among the five studied regions in Fukushima prefecture, nearly all sites exhibited a gradually decrease in each radiocesium isotope concentration during the three years (Figure 3). In the Fukushima, Koriyama and Aizu regions, the total Cs, ^134^Cs and ^137^Cs concentrations decreased significantly during the three years after the F1NPP accident (r = -0.349 to -0.630, F = 112-156, p < 0.0001 in the Fukushima region, r = -0.329 to -0.493, F = 68-146, and p < 0.005-0.0001 in the Koriyama region, and r = -0.168 to -0.385, F = 283-642, and p < 0.005-0.0001 in Aizu region) (Figure 3). In the Iwaki region, a significant negative correlation was found for the total cesium concentration (r = -0.418, F = 32, and p < 0.05), while no significant correlations were found for the ^134^Cs and ^137^Cs concentrations (F = 3-5 and p > 0.1) due to the limited number of samples that were examined (Figure 3). Between May 2011 and March 2012, most of the studied fish had Cs concentrations that exceeded 1000 Bq kg^-1^ (Figure 3) because the F1NPP site is located near this region.

**Figure 4:**
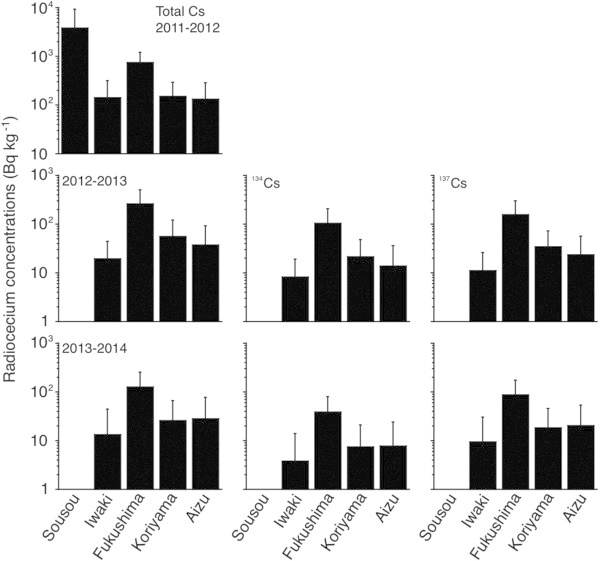
**Differences in radiocesium accumulations among the five regions between May 2011 and March 2014, i.e., after the F1NPP accident.** Between May 2011 and March 2012, the highest total Cs concentration was found in the Sousou region. The second highest concentration was found in the Fukushima region, which was followed by the Koriyama and Aizu regions. Significantly higher total Cs, ^134^Cs and ^137^Cs concentrations were found in the Fukushima region compared to the other regions between April 2012 and March 2013 and between April 2013 and March 2014.

The accumulation of each radiocesium isotope was different among the five regions in each year. In the Sosou, Iwaki, Fukushima, Koriyama and Aizu regions between May 2011 and March 2012, the total cesium concentrations ranged from 202 Bq kg^-1^ to 18700 Bq kg^-1^ (with a mean and standard deviation of 3809 ± 5389 Bq kg^-1^), nd to 720 Bq kg^-1^ (142 ± 172 Bq kg^-1^), 89 to 2080 Bq kg^-1^ (747 ± 454 Bq kg^-1^), nd to 620 Bq kg^-1^ (151 ± 140 Bq kg^-1^) and 0 to 870 Bq kg^-1^ (132 ± 153 Bq kg^-1^) (Figure 4). The highest concentrations were detected in the Sousou region compared to the other four regions (F = 9 and p < 0.0001) (Figure 4). The second higher concentrations were found in the Fukushima region, which was followed by the Koriyama and Aizu regions (F = 37-41 and p < 0.05-0.005); no significant differences were found between the Fukushima region and the Iwaki region (F = 47 and p > 0.05) (Figure 4). There were no significant differences found between other combinations (F =37-64 and p > 0.05) (Figure 4).

The total cesium concentrations between April 2012 and March 2013 ranged from nd to 65 Bq kg^-1^ (19.4 ± 24.5 Bq kg^-1^) in Iwaki region, nd to 1400 Bq kg^-1^ (261 ± 242 Bq kg^-1^) in the Fukushima region, nd to 250 Bq kg^-1^ (55.9 ± 63.9 Bq kg^-1^) in the Koriyama region and nd to 420 Bq kg^-1^ (37.3 ± 54.2 Bq kg^-1^) in the Aizu region (Figure 4). The ^134^Cs and ^137^Cs concentrations in these regions during the same period were nd to 25.5 Bq kg^-1^ (8.19 ± 10.8 Bq kg^-1^) and nd to 39.3 Bq kg^-1^ (11.1 ± 14.8 Bq kg^-1^), nd to 610 Bq kg^-1^ (104 ± 102 Bq kg^-1^) and nd to 820 Bq kg^-1^ (156 ± 143 Bq kg^-1^), nd to 100 Bq kg^-1^ (21.4 ± 26.3 Bq kg^-1^) and nd to 150 Bq kg^-1^ (34.4 ± 37.6 Bq kg^-1^) and nd to 171 Bq kg^-1^ (13.8 ± 22.1 Bq kg^-1^) and nd to 253 Bq kg^-1^ (23.5 ± 32.5 Bq kg^-1^), respectively (Figure 4). Significantly higher concentrations of total cesium, ^134^Cs and ^137^Cs were found in the Fukushima region compared with the other regions (F = 57 to 63 and p < 0.0001), while no significant differences were found between the other regions (F = 6 – 82 and p > 0.05) (Figure 4).

The total Cs, ^34^Cs and ^137^Cs concentrations between April 2013 and March 2014 ranged from nd to 82 Bq kg^-1^ (13.3 ± 30.6 Bq kg^-1^), nd to 26.6 Bq kg^-1^ (3.8 ± 10.1 Bq kg^-1^) and nd to 55.5 Bq kg^-1^ (9.4 ± 20.7 Bq kg^-1^) in the Iwaki region, from nd to 600 Bq kg^-1^ (126 ± 126 Bq kg^-1^), nd to 194 Bq kg^-1^ (38.7 ± 40.9 Bq kg^-1^) and nd to 403 Bq kg^-1^ (87.1 ± 85.5 Bq kg^-1^) in the Fukushima region, from nd to 180 Bq kg^-1^ (25.8 ± 40.1 Bq kg^-1^), nd to 56.3 Bq kg^-1^ (7.4 ± 13.4 Bq kg^-1^) and nd to 127 Bq kg^-1^ (18.3 ± 27.1 Bq kg^-1^) in the Koriyama region and from nd to 390 Bq kg^-1^ (28.1 ± 48.7 Bq kg^-1^), nd to 126 Bq kg^-1^ (7.7 ± 16.2 Bq kg^-1^) and nd to 261 Bq kg^-1^ (20.3 ± 32.9 Bq kg^-1^) in the Aizu region (Figure 4). Significantly higher total Cs, ^134^Cs and ^137^Cs concentrations were found in the Fukushima compared to the other regions (F = 35 to 74 and p < 0.0001), while no significant differences were found between the other regions (F = 6- 143 and p > 0.05) (Figure 4).

In the Iwaki, Fukushima, Koriyama and Aizu regions, the detectable rate of each radiocesium isotope decreased during the three years except for the Fukushima region (Table 3). Although a limited number of sample were examined for the Iwaki region between April 2012 and March 2014, the detectable rate for each radiocesium isotope decreased by less than 30% during the third year, which was less than the 80% reduction that was observed in the total cesium detection rate during the first year (Table 3). In the Koriyama and Aizu regions, the detectable rates for ^134^Cs decreased by 30% in the third year; the second year exhibited a decrease that exceeded 50% (Table 3). Regarding the total cesium and ^137^Cs in these regions, the detectable rates also decreased during years two and three (Table 3). However, such tendencies were not found in the Fukushima region. The detectable rates for the total cesium and ^137^Cs in the Fukushima region were constant more than 95% of the time 3 years of the F1NPP accident. Although the detectable rate for ^134^Cs decreased slightly between the second year and third year, the detectable rates in each year (98% in the second year and 86% in third year) clearly exceeded those for the other regions (Table 3). These results suggest that radiocesium inputs continue in the Fukushima region compared with the other regions in Fukushima prefecture.

**Table 3:** **Radiocesium data information in five regions in Fukushima prefecture examined between May 2011 and March 2014 in the present study**

Region	Number of analysed betweeen May 2011 and March 2012	Number of data above detection limit betweeen May 2011 and March 2012 (Ratio of detectable (%))			Number of analysed betweeen April 2012 and March 2013	Number of data above detection limit betweeen April 2012 and March 2013 (Ratio of detectable (%))			Number of analysed betweeen April 2013 and March 2014	Number of data above detection limit betweeen April 2013 and March 2014 (Ratio of detectable (%))		
		Total Cs*	^134^Cs	^137^Cs		Total Cs*	^134^Cs	^137^Cs		Total Cs*	^134^Cs	^137^Cs
Sousou region	10	10	n/a	n/a	n/a	n/a	n/a	n/a	n/a	n/a	n/a	n/a
		(100)										
Iwaki region	31	26	n/a	n/a	9	7	3	4	7	2	1	2
		(83.9)				(77.8)	(33.3)	(44.4)		(28.6)	(14.3)	(28.6)
Fukushima region	37	37	n/a	n/a	58	57	57	57	65	63	56	63
		(100)				(98.3)	(98.3)	(98.3)		(96.9)	(86.1)	(96.9)
Koriyama region	43	42	n/a	n/a	70	57	43	57	83	48	26	48
		(97.7)				(81.4)	(61.4)	(81.4)		(57.8)	(31.3)	(57.8)
Aizu region	210	188	n/a	n/a	541	369	278	364	358	230	117	228
		(89.5)				(68.2)	(51.3)	(67.3)		(64.2)	(32.7)	(63.7)

*Total Cs = ^134^Cs + ^137^Cs.

Total Cs was available between May 2011 and March 2014.

^134^Cs + ^137^Cs were available between April 2012 and March 2014.

n/a: not applicable.

### Accumulation patterns of radiocesium in freshwater fish species

**Figure 5:**
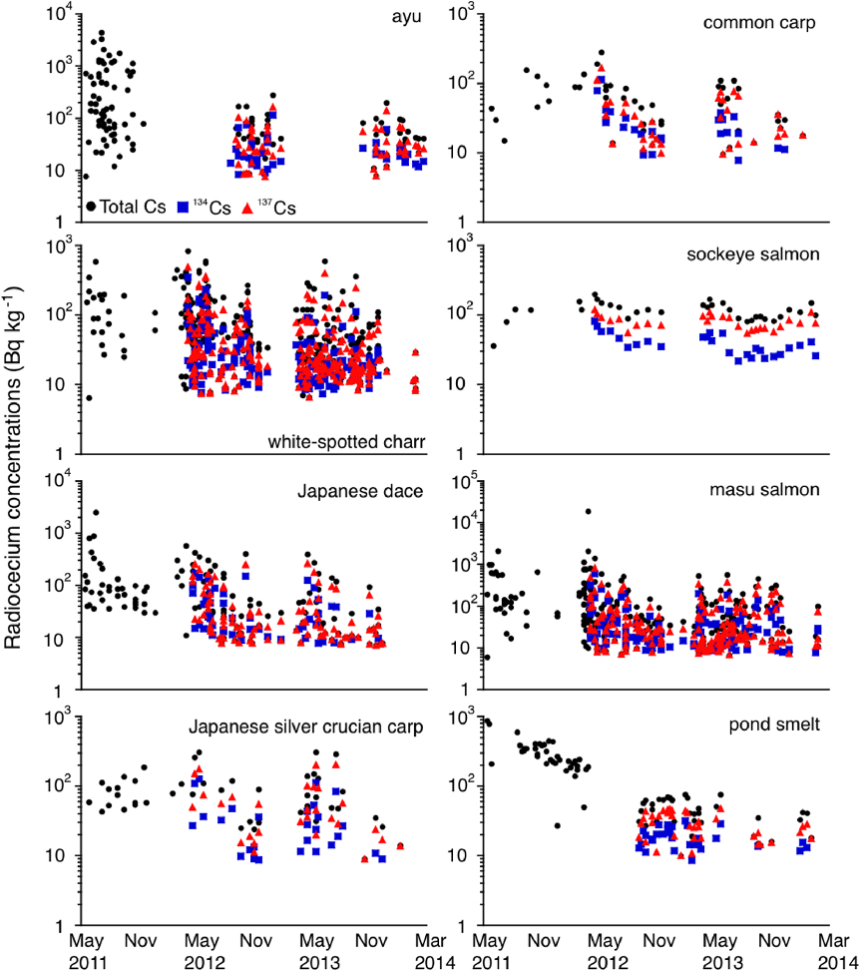
**Accumulation patterns of radiocesium in freshwater fish species for three years.** The highest concentration was found in masu salmon (18700 Bq kg^-1^). The second highest concentration was found in ayu (2900-4400 Bq kg^-1^), while the third highest concentration was found in Japanese dace (2500 Bq kg^-1^). All of the fish were collected in the Sousou region between May 2011 and March 2012. Although each radiocesium isotope concentration decreased significantly in most of the freshwater fish species, the tendencies varied among the species.

In the present study, the highest Cs concentration was found in masu salmon (18700 Bq kg^-1^). The second highest concentration was found in ayu (2900-4400 Bq kg^-1^), while the third highest concentration was found in Japanese dace (2500 Bq kg^-1^). All of these fish were collected in the Sousou region between May 2011 and March 2012 (Table 2 and Figure 5). In this study, eight of the 14 freshwater fish species could be examined throughout the three-year period because a sufficient number of samples was available (Table 2 and Figure 5). Although the most recent data did not exhibit concentrations exceeding 1000 Bq kg^-1^ or 10000 Bq kg^-1^ in freshwater species, concentrations exceeding 100 Bq kg^-1^, which is the safety threshold for the total Cs that was introduced in April 2012 in Japan, were detected (Table 2 and Figure 5).

Negative correlations between each radiocesium (i.e., total Cs, ^134^Cs , ^137^Cs) concentration and the elapsed time were found in ayu (F = 38-120 and p < 0.0001), white-spotted char (F = 156-282 and p < 0.0001) and Japanese dace (F = 48-136 and p < 0.05-0.0005) (Figure 5). Negative relationships were found between the total Cs and ^134^Cs concentrations and the elapsed time (F = 25-45 and p < 0.05), while no correlation was found for the ^137^Cs concentration (F = 34 and p > 0.1) in common carp (Figure 5). In sockeye salmon, a negative relationship was found between the ^134^Cs concentrations and the elapsed time (F = 26 and p < 0.0001), while no correlations were found for the total Cs and ^137^Cs concentrations (F = 26-32 and p > 0.1-0.5). Moreover, the ^134^Cs and ^137^Cs concentrations decreased significantly during the three years (F = 118-177 and p < 0.05), while no relationship was found for the total Cs concentration (F = 240 and p > 0.05) in masu salmon. Furthermore, nearly the same negative relationship was found between the total Cs and ^137^Cs concentrations and the elapsed time (F = 36-75 and p < 0.05-0.0001), while no correlation was found for the ^134^Cs concentration (F = 30 and p > 0.1) in Japanese smelt. No negative relationships were found between the total Cs, ^134^Cs and ^137^Cs concentrations and the elapsed time in Japanese silver crucian carp (F = 26-44 and p > 0.1-0.5). Although each radiocesium concentration decreased significantly in most of the studied freshwater fish species, the tendencies varied among the species (Figure 5).

## Discussion

The present study showed that radiocesium accumulation gradually decreased during the three years after the F1NPP accident in Fukushima prefecture. However, concentrations exceeding 100 Bq kg^-1^, which is the interim limit for radiocesium level in Japan, were detected in several fish species (Figure 2). Radioactive accumulation in freshwater fish occurs when the fish retain contaminated materials from freshwater environments. The results of this study also suggest that radiocesium accumulation in terrestrial environments in the Fukushima regions decreased gradually after the accident. However, brown trout in a Norwegian lake that was contaminated by radiocesium fallout from the Chernobyl accident still contain radiocesium accumulations more than 20 years after the accident; these concentrations are thought to be no longer decreasing (Brittain and Gjerseth 2010). This phenomenon might be due to the continual input of radiocesium substances from the catchment and remobilization in the local terrestrial environment. Therefore, continuous long-term monitoring of radioactive accumulation in fish is needed to understand the fate and effects in terrestrial environments and to evaluate the aftermath of the F1NPP accident. Because fish are major structural and functional components in freshwater ecosystems, radioactive contamination of fish should be avoided because they are consumed by terrestrial animals and human. Sporadically detected accumulations exceeding the interim limit for foods in the Fukushima regions were detected even three years after the F1NPP accident. The Japanese interim limit for imported foods was 370 Bq kg^-1^ for the total Cs concentration after the Chernobyl nuclear accident in 1986. The interim limit after the F1NPP accident was initially set at 500 Bq kg^-1^, however stricter limit of 100 Bq kg^-1^ was introduced in April 2012. A tissue analysis indicated that 75% of the total ^137^Cs accumulated in the flesh of the examined fish, i.e. the edible parts of the fish (Malek et al. 2004). This result indicated that most of the consumed ^137^Cs by fish was deposited in soft tissue. Therefore, as long as radiocesium that exceed the interim limit are detected in fish, commercial fisheries and consumption of fish from these regions should be prohibited in the Fukushima regions.

Radiocesium accumulation patterns in fish in Fukushima prefecture differed among the studied regions. The highest accumulation of total Cs was found in the Sousou region, which is where the F1NPP site is located; the accumulations tended to decrease as a function of the distance from the F1NPP site (Figure 4). However, the radiocesium accumulations in the Fukushima region were significantly higher than those in the Iwaki and Koriyama regions despite these regions being located adjacent to the Sousou region (Figure 4). The spatial accumulation patterns of radiocesium found in the present study represent well-documented natural radiocesium levels. The deposition of radiocesium on the land surrounding the F1NPP site was evident due to the higher levels that were detected in northwest of the FlNPP site (Figure 1B). Date city and Kawamata town are associated with the Fukushima region; higher total Cs concentrations (from 10^6^ to 30^6^ Bq m^-3^) were detected in these areas compared to the surrounding locations in April 2011; the concentrations in the Iwaki and Koriyama regions were less than 30^5^ - 60^5^ Bq m^-3^ (Figure 1B). The deposition patterns of rediocesium might correspond to the atmospheric circulation; therefore environmental radiocesium levels in the Fukushima region were higher than in the Iwaki and Koriyama regions, which caused the higher accumulation of radiocesium in fish in the Fukushima region. The detectable rate of each radiocesium isotope concentration was much higher in the Fukushima region, i.e., 97-100% for the total Cs and ^137^Cs concentration and 86-98% for the ^134^Cs concentration throughout the three-year period between May 2011 and March 2014. However, the detectable rates in the Iwaki and Koriyama regions decreased to 14-29% and 31-58%, respectively (Table 3). These results suggest that radionuclides remain in the natural environment with less convergence. Moreover, there is a continual input of radiocesium substances from the F1NPP site into the local terrestrial environment.

Radiocesium accumulations in freshwater fish species examined in the present study might represent the spatial accumulations of these radionuclides. Arai (2014) found that salmon migration patterns revealed the temporal and spatial variations in radiocesium concentrations in terrestrial and oceanic environments. In the present study, three salmon species, i.e., white-spotted char, sockeye salmon and masu salmon, were examined. The accumulation trends of radiocesium during the studied three-year period in these salmon species were similar to those found for all freshwater fish species (Figures 2 and 5). Because these salmon species, especially the white-spotted char and masu salmon are common species and are distributed over a broader region in Japan and other East Asian countries, these salmon species may be good indicators for tracing the environmental radiocesium levels after the F1NPP accident.

Although the higher levels of radiocesium in freshwater fish are caused by the serious contamination in the terrestrial environments surrounding Fukushima prefecture after the F1NPP accident, freshwater fish exhibit ^137^Cs concentrations that are approximately 100 times higher than those of marine fishes due to the presence of potassium, which is less abundant in freshwater. This difference results in the accelerated uptake of cesium by freshwater organisms (Harte et al. 1991). Thus, the differences in the physiological mechanisms, especially the ionic uptake and elimination of the related osmoregulation, might also accelerate ^137^Cs accumulation in freshwater fish. The F1NPP accident released large amounts of radioactive substances into the environment and contaminated the soil of the Tohoku and the Kanto (East) districts in Japan. The radiocesium concentrations in the water, soil and biota in the terrestrial environment are much higher than those of the coastal and marine environments (Yasunari et al. 2011; Buesseler et al. 2012; Arai 2014). The natural habitats of freshwater fish vary among species. Although fish reside in streams, ponds and lakes, several species have restricted home ranges. In the present study, the accumulation trends of radiocesium as a function of the elapsed times were different among the studied species (Figure 5). Many studies have analysed the accumulation and loss of radiocesium in freshwater fishes (Meili 1991; Ugedal et al. 1992; Forseth et al. 1992; Morgan et al. 1993; Rowan and Rasumussen 1995; Peters et al. 1999; Malek et al. 2004). There are several ways in which fish can take up radionuclides: (1) uptake through a contaminated diet in which the food is contaminated due to contact with contaminated water or by food chain intake (2) absorption through the skin, and (3) absorption through the gills (Malek et al. 2004). The present study suggests that freshwater fish may uptake radiocesium primarly from contaminated diets; however, the contamination levels vary according to the environmental radiocesium levels.

In freshwater ecosystems, the initial dynamic phase of ^137^Cs contamination and equilibration after a fallout lasts up to five years and appears to be largely determined by biological processes (Bergan 1995). Thereafter, ^137^Cs activities in fish approach a steady state, with a slow decrease that is likely controlled by continuous secondary inputs of ^137^Cs into freshwater environments such as streams, lakes and ponds and their food webs (Bergan 1995). The most likely sources are the loss of ^137^Cs from land to water and the recycling of ^137^Cs from sediments. While fluxes and abiotic concentrations of ^137^Cs decrease, the bioavailability of ^137^Cs in freshwater ecosystems continually decreases (Bergan 1995). However, in regard to the F1NPP accident, only three years have passed. Therefore the initial dynamic phase of contamination and equilibration may be still continuing and unstable. However, the present study has suggested that the accumulation of radiocesium has gradually decreased in freshwater fish in the Fukushima regions. Monitoring the long-term behaviour of radionuclides in the environment is an important issue for estimating possible radiological consequences and associated risks. This monitoring is also important for evaluating the potential use of contaminated areas and the possible effectiveness of remediation activities. Further continuous monitoring is indispensable for understanding radionuclide behaviour in freshwater ecosystems and human health after the F1NPP accident.
